# Volatile oils of Chinese crude medicines exhibit antiparasitic activity against human *Demodex* with no adverse effects *in vivo*

**DOI:** 10.3892/etm.2015.2272

**Published:** 2015-02-06

**Authors:** JI-XIN LIU, YAN-HONG SUN, CHAO-PIN LI

**Affiliations:** 1 Department of Clinical Pathogen Biology, Qiqihar Medical University, Qiqihar, Heilongjiang 161006, P.R. China; 2 Department of Medical Parasitology, Wannan Medical College, Wuhu, Anhui 241002, P.R. China

**Keywords:** Chinese crude medicine, volatile oil, *Demodex*, anti-*Demodex* activity

## Abstract

*Demodex* is a type of permanent obligatory parasite, which can be found on the human body surface. Currently, drugs targeting *Demodex* usually result in adverse effects and have a poor therapeutic effect. Thus, the aim of the present study was to investigate the use of Chinese crude medicine volatile oils for targeting and inhibiting *Demodex in vitro*. The volatile oils of six Chinese crude medicines were investigated, including clove, orange fruit, Manchurian wildginger, cinnamon bark, Rhizome Alpiniae Officinarum and pricklyash peel, which were extracted using a distillation method. The exercise status of *Demodex folliculorum* and *Demodex brevis* and the antiparasitic effects of the volatile oils against the two species were observed using microscopy. A skin irritation test was used to examine the irritation intensity of the volatile oils. In addition, an acute toxicity test was utilized to observe the toxicity effects of the volatile oils on the skin. Xin Fumanling ointment was employed as a positive control to identify the therapeutic effects of the volatile oils. The results indicated that all six volatile oils were able to kill *Demodex* efficiently. In particular, the clove volatile oil was effective in inducing optimized anti-*Demodex* activity. The lethal times of the volatile oils were significantly decreased compared with the Xin Fumanling ointment (P<0.05). Furthermore, the skin irritation test results indicated that the clove volatile oil did not trigger any irritation (0.2 and 0.3 points for intact and scratched skin, respectively), and had a safety equal to that of distilled water. There were not any adverse effects observed following application of the clove volatile oil on the intact or scratched skin. In conclusion, the volatile oils of Chinese crude medicines, particularly that of clove, demonstrated an evident anti-*Demodex* activity and were able to kill *Demodex* effectively and safely *in vivo*.

## Introduction

*Demodex*, a type of permanent obligatory parasite (class, Arachnida; superorder, Acariformes), is an elongated ectoparasite found on the human body surface, including the face, cheeks, forehead, nose and eyelids (1). There are numerous species of *Demodex*, but only *Demodex folliculorum* and *Demodex brevis* are found on the human body (2,3). The organism, *Demodex folliculorum,* is found in the eyelash follicle, while *Demodex brevis* burrows deep into the sebaceous and meibomian glands (3). The rate of *Demodex* infestation increases with age, with the organism identified in 84% of the population at age 60 and in 100% of those aged >70 years (4). In addition to the higher density of *Demodex* observed in patients with rosacea (5), *Demodex* has also been suggested as a cause of other skin diseases, including pityriasis folliculorum, perioral dermatitis (6,7), scabies-like eruptions, facial pigmentation, eruptions of the bald scalp, demodicosis gravis and even basal cell carcinoma (8,9). Although the pathogenic role remains elusive, efforts have been made to eradicate ocular *Demodex* in patients presenting with blepharitis (10).

To date, a number of studies (6,7,10) have investigated various drugs targeting *Demodex*; however, the majority of these drugs were not found to be effective. There are seldom studies investigating the antiparasitic effects of volatile oils from Chinese crude drugs against *Demodex*. Thus, the present study selected six types of Chinese crude drugs, among the 300 drugs that are rich in volatile oil. Subsequently, the anti-*Demodex* efficacy and safety were studied for the six volatile oils used in the *Demodex* therapy.

## Materials and methods

### Materials, reagents and animals

Clove, orange fruit, Manchurian wild ginger, cinnamon bark, Rhizoma Alpiniae Officinarum and pricklyash peel were purchased from Huainan Hengkang Medicine Co., Ltd. (Huainan, China). The quality of these drugs was assessed by Professor Genbao Zhang in the Department of Pharmacy (Qiqihar Medical University, Qiqihar, China). An optical microscope and photographic system were purchased from Nikon Corporation (model SMZ1500, Tokyo, Japan). The clinical anti-*Demodex* drug, Xin Fumanling ointment, was purchased from Shandong Jiankang Pharmaceutical Co., Ltd. (Jinan, China). In total, four male rabbits and four female rabbits (weight, 2.0–2.5 kg) were provided by the Experimental Animal Base of Anhui University of Science and Technology (Huainan, China). The eight rabbits were divided into two groups randomly; one group received treatment with the volatile oils, while the other group received Xin Fumanling ointment treatment. Furthermore, sodium chloride solution was selected as the negative control. The present study was approved by the Ethics Committee of Qiqihar Medical University.

### Extraction of volatile oil

In total, 50-g samples of each Chinese crude medicine were smashed, filtered and soaked for 8 h. The extraction of volatile oil was performed using a distillation method, followed by drying with sodium sulfate. The detailed procedures were performed according to previous studies (11,12).

### In vitro experiment

Since there is a high prevalence of *Demodex* in patients with cylindrical dandruff, lashes with cylindrical dandruff were selected for the *in vitro* experiment (13). A total of 20 patients were selected to obtain the *Demodex*, these patients were diagnosed by the Department of Dermatology (Qiqihar Medical University). Informed consent was obtained from all of the patients. *Demodex* was obtained with cellulose tape at room temperature (30±0.5°C). The experiment was performed in a good manufacturing practice laboratory. Volatile oil was dripped onto the *Demodex* with a micropipette, and the movements of the *Demodex* body and legs were observed continuously under a microscope (CX31; Olympus, Tokyo, Japan) at ×40 magnification for 60 min. Death of the parasite was defined as no movements of the body and no changes to the legs for a minimum of 30 sec. The lethal time was defined as the time between the addition of the solution and when the organism stopped moving. Xin Fumanling ointment was used as the positive control, while saline served as the blank control.

### Skin irritation test

In total, a 0.3-μl sample of each volatile oil was applied to a section of the rabbit skin with the size of 2.5×2.5 cm. The skin was fixed with the bandage. Exposure time to the volatile oil lasted for 24 h, after which fresh saline was applied to the same site for clearing of the retained volatile oils.

### Measurements of skin irritation

Assessments of skin irritation were performed according to two criteria (14). The first criteria was scored as follows: No erythema, 0 points; speckling, moderate erythema, 1 point; uniform, moderate erythema, 2 points; intense erythema, 3 points; and intense erythema (red hot) with edema, 4 points. The second criteria was scored as follows: No edema, 0 points, extremely slight edema, 1 point; slight edema, 2 points; moderate edema, 3 points; and serious edema, 4 points. The skin irritation score was evaluated by combining the two criteria. No irritation was defined as 0.0–0.4 points, slight irritation was classified as 0.5–1.9 points, moderate irritation was defined as 2.0–5.9 points and intense irritation was classified as 6.0–8.0 points.

### Acute toxicity test

Acute toxicity of the volatile oils was investigated using the rabbits treated with *Demodex*. Observations of the rabbits were conducted for one week continuously. The aim of this experiment was to observe the systemic toxicity and mortality of the rabbits, including changes to the skin, hair, eyes and mucosa, changes to the respiratory system, circulatory system and central nervous system, limbs activities and behavioral style. In particular, observations of a tremble and muzziness were recorded. The criteria used for assessing acute toxicity was performed according to previous studies (15,16).

### Statistical analysis

Data are expressed as the mean ± standard deviation, and were analyzed using Microsoft Excel (Microsoft, Redmont, WA, USA). The data between groups were evaluated using a two-tailed t-test, where P<0.05 was considered to indicate a statistically significant difference.

## Results

### Antiparasitic activity of the volatile oils

**Figure 1:**
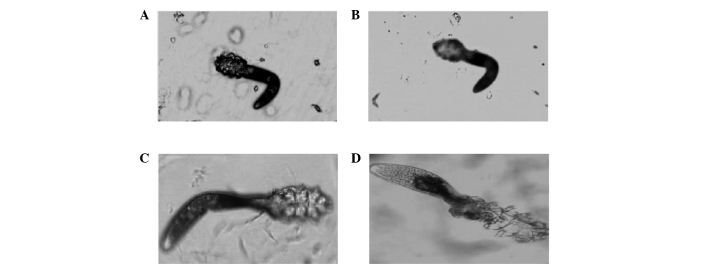
Mechanism underlying the antiparasitic process of the clove volatile oil on *Demodex folliculorum*. (A) Prior to administration of the volatile oil; (B) following full contact of *Demodex* with the volatile oil; (C) *Demodex folliculorum* appears marcid; (D) transparent body of *Demodex folliculorum*.

Sodium chloride was used as a negative control and was shown to have no effects on the rabbits (data not shown). Prior to the addition of the volatile oils, the two species of *Demodex* were active, with the limbs maintaining an exercise status (Fig. 1). The average exercise frequency of each limb was >15 times/min. Following addition of the volatile oils, the polypides exercised intensively and contracted, deforming significantly (Fig. 1). The exercise frequency significantly increased to >20 times/min. The *Demodex* polypide was exercising in the volatile oils, experiencing a shortly excited state, which indicated an inhibitory state. Finally, the activity of *Demodex* decreased significantly, and a parasiticide outcome was achieved.

### Lethal times of the volatile oils on Demodex

**Figure 2:**
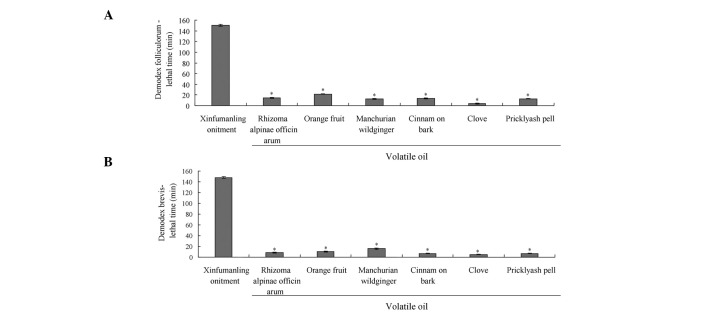
Lethal times of the six volatile oils on (A) *Demodex folliculorum* and (B) *Demodex brevis*. ^*^P<0.05, vs. Xin Fumanling ointment.

All six types of volatile oils were shown to kill the two species of *Demodex* within 30 min. All the volatile oils demonstrated a significantly higher antiparasitic activity compared with the positive control of Xin Fumanling ointment (Fig. 2; P<0.05). In addition, the antiparasitic activity of the volatile oil from Manchurian wild ginger and clove on *Demodex folliculorum* were significantly higher compared with that on *Demodex brevis* (Fig. 2; P<0.05).

### Volatile oils trigger no significant skin irritation

**Figure 3:**
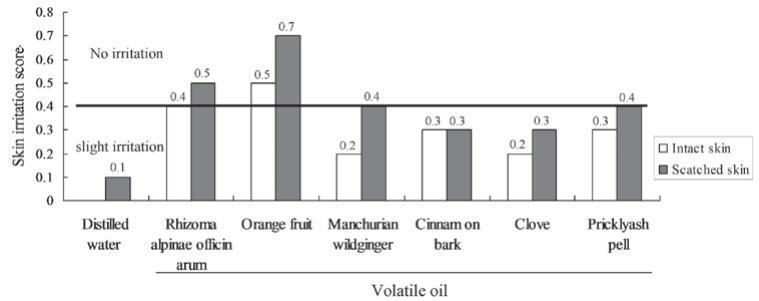
Skin irritation scores of the intact skin and scratched skin treated with the various volatile oils. ^*^P<0.05, vs. distilled water. Black line indicates the division between no irritation (scores of 0–0.4) and slight irritation (scores of >0.4).

Results from the skin irritation test indicated that Rhizoma Alpiniae Officinarum and orange fruit induced only a slight irritation. Furthermore, Manchurian wild ginger, cinnamon bark, clove and pricklyash peel were not shown to induce any irritation for the intact skin or scratched skin, with scores of <0.4 points (Fig. 3). In particular, the clove volatile oil was not found to trigger any irritation, with effects equivalent to those of distilled water.

### No significant adverse effects were observed with the volatile oil-treated skin

**Figure 4:**
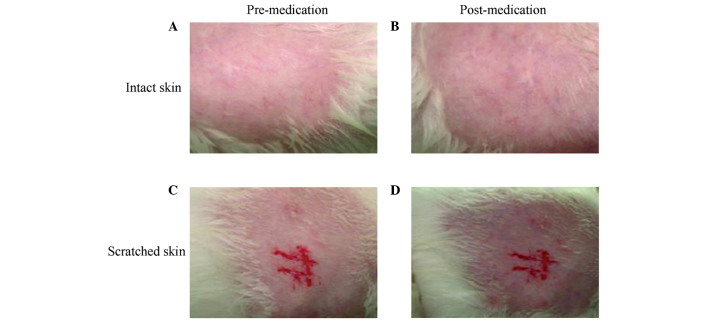
Effects of the clove volatile oil on the rabbit skin. (A) Intact skin prior to administration; (B) intact skin following administration; (C) scratched skin prior to administration; and (D) scratched skin following administration.

After one week of observation, no adverse effects were observed on the intact skin and scratched skin of the rabbits. The weight, hair, skin, respiratory system and limb activity of the rabbits were not shown to exhibit a significant abnormality (Fig. 4).

## Discussion

A number of drugs have been applied as anti-*Demodex* therapy, including 1–2% metronidazole (17), 2 or 5% permethrin (18), 200 g/kg ivermectin (19), amongst others. However, the aforementioned drugs require complex synthetic technology, and have been shown to exert certain adverse effects following application. Chinese medicine shares the benefits of reduced side effects, minor toxicity and few side effects; thus, may play an important role in anti-*Demodex* therapy. To date, a number of Chinese drugs have been synthesized for *Demodex* therapy in China.

Volatile oil is a type of oily liquid with characteristics of extensive bioactivity and an aromatic odor (20). The present study selected six types of volatile oils, extracted using a distillation method, and observed their anti-*Demodex* activity. The results indicated that all the volatile oils possessed more intense anti-*Demodex* activity compared with the positive control. Among these volatile oils, the clove volatile oil provided the most intense anti-*Demodex* activity.

A previous study (21) also investigated the effects of clove, orange fruit, Manchurian wildginger, cinnamon bark, Rhizome Alpiniae Officinarum and pricklyash peel. However, the anti-*Demodex* function has not yet been fully explored. In the present study, clove volatile oil was adopted as the therapeutic drug, which was shown to trigger significant anti-*Demodex* activity compared with the Xin Fumanling ointment. According to the criteria outlined by the National Health and Family Planning Commission of China (Beijing, China), all maquillage and drugs designed for the skin should not cause mucosal irritation and damage. In the present study, a skin irritation test was performed to identify the safety of the volatile oils. From the irritation test results, it was found that no erythema or edema were observed in the intact skin, and only slight erythema was observed in the scratched skin following the application of clove volatile oil. An acute toxicity test was applied to analyze the toxicity and adverse effects. The results revealed that the clove volatile oil did not exhibit acute toxicity on the rabbit skin. Clove resources are very rich, and the volatile oil can be extracted conveniently. The low toxicity and safety demonstrate the feasibility of using clove volatile oil in clinical and basic studies.

In conclusion, Chinese crude medicine volatile oil, particularly that for clove, demonstrated a marked anti-*Demodex* activity. The clove volatile oil was shown to kill *Demodex* effectively and safely *in vivo*.
